# Multivariable prediction of early malignancy detection among first-degree relatives of patients with hereditary colorectal cancer based on the health belief model: A cross-sectional study from the largest hereditary colorectal cancer cohort in China

**DOI:** 10.1371/journal.pone.0323369

**Published:** 2025-05-16

**Authors:** Liyuan Yang, Xiaojun Chen, Mengjiao Zhong, Zhiyong Pan, Xiaodan Wu

**Affiliations:** 1 Guangzhou Women and Children's Medical Center, Guangzhou Medical University, Guangzhou, People's Republic of China; 2 School of Economics and Management, Beijing University of Posts and Telecommunications, Beijing, China; 3 Sun Yat-sen University Cancer Center; State Key Laboratory of Oncology in South China; Collaborative Innovation Center for Cancer Medicine, Guangzhou, People's Republic of China; 4 Department of Neurology, Yueyang People’s Hospital, Hunan Normal University, Yueyang, China; 5 Unit of Psychiatry, Institute of Translational Medicine, Faculty of Health Sciences, University of Macau, Macao SAR, China; The University of Sheffield, UNITED KINGDOM OF GREAT BRITAIN AND NORTHERN IRELAND

## Abstract

**Background:**

First-degree relatives (FDRs) of hereditary colorectal cancer are at an increased risk of cancer and receiving early detection and surveillance on malignancies is an efficacious strategy to reduce cancer-related morbidity and mortality. But there is rare information on screening in this cancer high-risk group in China.

**Objective:**

The aim of this study was to explore the detection and surveillance on malignancies and its predictors among FDRs of patients with hereditary CRC based on the largest hereditary colorectal cancer cohort from China.

**Methods:**

We conducted an exploratory, descriptive, cross-sectional study. 530 FDRs were recruited from December 2021 to December 2022, evaluated using the questionnaire on knowledge, attitudes and behavior of malignancies early detection and the Champion’s Health Belief Model Scale. The main outcome were identified using logistic regression analysis.

**Results:**

Among all the FDRs, only 122 (23.0%) underwent malignancies early detection. The predictors of malignancies early detection included knowledge score (*OR* = 1.117, *P < *0.001), sex (*OR* = 0.244, *P < *0.001), age (*OR* = 4.627, *P < *0.001), marital status (*OR* = 3.815, *P < *0.001), chronic disease history (*OR* = 2.945, *P < *0.01), diagnosis of index patient (*OR* = 2.876, *P < *0.001), attitude about “cancer is preventable” (*OR* = 3.405, *P < *0.05) and “one needs malignancies early detection even if feel healthy” (*OR* = 16.477, *P < *0.001), perceived susceptibility (*OR* = 1.106, *P < *0.05) and self-efficacy (*OR* = 1.244, *P < *0.001).

**Conclusion:**

The uptake of malignancy early detection among FDRs should be improved. Some demographic and health-related characteristics, knowledge score, perceived susceptibility and self-efficacy were the most important predictors of malignancies early detection. Enhancing the recognition of clinical features of hereditary CRC and offering personalized genetic counseling, along with tailored cancer risk assessments, could further optimize individual cancer surveillance and prevention strategies, helping to reduce the risk of malignancies in high-risk populations.

## Introduction

Colorectal cancer (CRC) has become the third most common cancer and the second leading cause of cancer-related deaths worldwide [1,2]. According to GLOBOCAN 2021, nearly 2 million new cases and 1 million deaths occurred, accounting for 10.0% of new cancer cases and 9.4% of all cancer-related deaths [2]. In China, CRC is the second most common malignancy, with the incidence rising annually. Each year, there are approximately 408,000 new cases and 196,000 deaths [1]. The 5-year survival rate for patients diagnosed early with CRC is over 90%, while for those diagnosed at advanced stages, it drops below 10% [3]. Unfortunately, 27.6–51.1% of CRC patients are diagnosed at advanced stage [3]. Xiaodan Wu’s study found that over 47% of Chinese CRC patients delay diagnosis and treatment by at least three months, which worsens the cancer stage upon diagnosis [4].

Approximately 30% of individuals with CRC have a family history of the disease, and a subset of these patients are affected by hereditary colorectal cancer, accounting for more than 3% of all CRC patients [5]. Hereditary colorectal cancer refers to colorectal cancer resulting from genetic factors, such as specific gene pathogenic variants, which are often inherited within families. These pathogenic variants notably increase the risk of cancer in close relatives, and may be associated with various genetic syndromes, mainly Lynch syndrome or familial adenomatous polyposis (FAP). From a clinical perspective, individuals with hereditary CRC characteristics are at risk for early-onset cancer, often diagnosed before the age of 45. They also face a high lifetime risk—ranging from 40% to 90%—of developing multiple primary malignancies. These include a predisposition to colorectal tumors, as well as frequent occurrences of multiple primary tumors in the same patient, along with an increased risk of other related cancers, particularly in the ovary, endometrium, small bowel, and urothelium [5]. Hereditary CRC is the most common forms of hereditary cancer [6]. First-degree relatives (FDRs) (parents, children, and siblings) of patients with CRC have a 50% chance of carrying the same pathogenic variant in conditions such as Lynch syndrome [6], which is associated with accelerated progression of precancerous adenomas in colorectum to carcinomas and other relative cancer. Therefore, the FDRs of hereditary CRC patients have a six- to eight-fold increased risk of relative cancer than the general population.

As is well-known, cancer screening could improve the therapeutic effect, reduce morbidity and mortality, and save treatment costs and health resource by detecting and removing precancerous adenomas and by detecting carcinomas at an early, curable stage [7]. Colonoscopy provides an opportunity for early intervention of hereditary CRC. In a cohort of population with hereditary CRC pathogenic genes from Finnish who were offered colonoscopies surveillance every 2 years, the incidence of CRC and CRC-related mortality was reduced by at least 63% and 66% respectively [8,9]. Therefore, the US Multi-Society Task Force on Colorectal Cancer recommends that FDRs of hereditary CRC patients undergo biennial colonoscopy surveillance starting at ages 20–25, or approximately 10 years before the youngest age of onset in the family [10,11]. Furthermore, due to the rising risk of endometrial and other cancer, guidelines recommend that the FDRs of patients with hereditary CRC undergo gastroscopy, endometrial detection, and other relative inspection [6].

However, the acceptance of early detection and surveillance for malignancies in FDRs of individuals with CRC worldwide remains very low, at 22.2% to 60% [12,13]. On the basis of one study from a representative cancer center in China, only 20.9% individuals accepted colonoscopy as recommended [14]. For general population, one important criterion for an effective cancer screening program is that at least 70% of the target individuals complete the detection [15]. For the high risk population, especially the FDRs of individals with hereditary CRC, the criteria of effective early detection and surveillance for malignancies should be relatively higher. It is a highly targeted, efficient and simple method for preventing and controlling cancer to improving malignancies early detection among FDRs. Some studies in foreign countries have suggested that many FDRs of hereditary CRC, do not have malignancies early detection as recommended by guidelines, some are never underwent cancer surveillance at all [16]. As is well-known, there is limited information on early detection for malignancies and its related factors in the FDRs of ones with hereditary CRC in Asian area.

There are many factors could influence adherence of early detection and surveillance for malignancies. Some scholars [13] found that age was one of the most important predictor of screening. In addition, ones with a health insurance and higher educational level reported higher possibility of receiving early detection and surveillance for malignancies [17]. Other factors that affect early detection include an individual’s knowledge about the disease, health beliefs, and health behaviors. These may include a poor understanding of guidelines for early detection, insufficient health promotion efforts by medical professionals, and barriers such as fear of colonoscopy and the high cost of the procedure [18]. However, few studies indicated predictors of early detection for malignancies among FDRs of patients with hereditary CRC, particularly in China.

Currently, there are few studies exploring the characteristics and predictors of early detection and surveillance for malignancies among FDRs of patients with hereditary CRC in China. Therefore, it is crucial to identify the factors that hinder early detection in this high-risk group, so that more effective interventions can be developed to promote the implementation of early detection strategies. The objectives of this study were to: (1) assess early detection practices, knowledge of colorectal cancer (CRC), attitudes toward early detection, and health beliefs among FDRs of individuals with hereditary CRC in China; (2) examine the disease-related and sociodemographic factors associated with early detection; and (3) identify the predictors of early detection for malignancies.

## Methods

### Setting and sample

A cross-sectional descriptive study was conducted from December 2021 to December 2022. Purposive sampling strategy was adopted in one first-class tertiary hospital in Guangzhou, South China. This hospital was chosen because it has the largest known cohort of hereditary colorectal cancer patients and represents the highest level in the field of hereditary cancer in China. The sample size was calculated based on 15% [19] (ϖ) of the prevalence rate of early detection for malignancies using a formula N=(Z^2^_α/2_ × ϖ×(1-ϖ))/δ^2^ with 0.03 allowable error (δ) and Z_α/2_ = 1.96 at 95% CI. Considering the 10% nonresponse rate, the final sample size was 599. All participants provided written informed consent.

### Participants

In this study, we focused on FDRs of patients with hereditary colorectal cancer. A total of 561 FDRs were recruited. The inclusion criteria were as follows: (1) FDRs (parents, siblings, or children) of patients diagnosed with hereditary colorectal cancer through genetic testing; (2) aged 18–70 years (according to the American College of Physicians, adults at high risk for hereditary CRC are likely to have related cancers between their 20s and 70s); (3) education level of at least primary school; (4) Chinese reading ability and proficiency in Mandarin communication; (5) willingness to participate in the study. Additionally, we included two types of FDRs: (1) those who underwent targeted genetic testing and received positive results for pathogenic variants; (2) those who chose not to undergo genetic testing. FDRs who tested negative for pathogenic variants were excluded, as they do not require intensive surveillance compared to the general population. Individuals with a history of malignant tumors or mental illness were also excluded.

It is important to note that we did not collect data on the specific hereditary cancer predisposition syndromes involved (e.g., Lynch syndrome, FAP, or other rarer syndromes). At the time of participant recruitment, our primary focus was to investigate FDRs’ behaviors and beliefs regarding early cancer detection, rather than conducting syndrome-specific analysis. However, we acknowledge this as a significant limitation, as different syndromes are associated with distinct cancer risks and screening recommendations. Future studies should incorporate detailed molecular or clinical classifications to enable more targeted and syndrome-specific research.

### Instruments

#### Demographic and health-related characteristics questionnaire.

Demographic and health-related characteristics of patients and FDRs were collected using self-reported questionnaires. The questionnaire was designed based on a literature review and included questions about sex, educational level, age, occupation, residence, marital status, family income, relationship with the index case, presence of other relatives with CRC, and history of chronic diseases. Additionally, information about the patient’s sex, age, cancer history, and diagnosis was also collected.

#### Questionnaire on knowledge, attitudes and behavior of malignancies early detection.

This questionnaire contained three sections, knowledge, attitudes and behavior of early detection and surveillance for malignancies, which were about hereditary CRC and early detection and surveillance for malignancies. It was revised based on the Chinese University of Hong Kong’s Colorectal Cancer Knowledge Questionnaire [20] with some contents simplified to suit hereditary CRC according the American Cancer Society and China Anti-Cancer Association. **Knowledge:** The knowledge part of the questionnaire included 3 aspects: disease symptoms, disease-related factors, screening and diagnostic methods, where options included 9 symptoms, 12 factors related to relative cancer of hereditary CRC and 6 main examination methods. Respondents can choose multiple answers for each section, and the score was 0 point for each wrong answer or ‘don’t know’ and 1 point for the correct answer, which was a total score of 27 points. **Attitudes:** This part was in regard to attitudes towards screening on cancer, for instance, early cancer is more likely curable, screening can help to detect CRC early, screening can help to prevent cancer, CRC is preventable, malignancies early detection is significant, malignancies early detection is advantage fo health, willingness to self-finance screening. The possible responses were ‘yes’ or ‘no’.**Assessment of behavior:** In particular, data on the actual practice of FDRs in undergoing malignancies early detection were collected using the question “Have you ever participated in malignancies early detection before?”; FDRs were segmented into “ever” and “never” screening on the basis of their reply. Respondents who answered ‘never’ were requested to select the reasons for not completing malignancies early detection. The ones answered “ever” should go on answering the questions “number of times malignancies early detection had been received” and “what examines (colonoscopy, FOBT, gastroscopy, screening for endometrial cancer, Ultrasound B, blood tumor marker and any other examines) had been received and the outcome”. The reliability was assessed by pretesting 80 individuals. The internal consistency of this questionnaires was implemented by calculating the Cronbach’s alpha based on the recommendation of > 0.70. The Cronbach’s alpha estimated was 0.811 and a test-retest reliability of 0.748. Finally, this questionnaire was modified and re-evaluated to fit the population of this study on the basis of feedback from the pretesting study.

#### Champion’s Health Belief Model Scale (CHBMS).

The CHBMS were developed by Jacobs based on the Champion’s Health Belief Model to assess the health beliefs of the population [18]. We have translated it and verified the validity and reliability before [21].This scale contained 6 dimensions: perceived severity (7 items), perceived susceptibility (5 items), perceived benefits (6 items), perceived barriers (6 items), health motivation (7 items) and self-efficacy (5 items). All items are rated based on a five-point Likert-type scale ranging from 1 (completely disagree) to 5 (completely agree) and the items of perceived barrier dimension are scored reversely. The higher is the score, the higher are the patient’s beliefs in health behavior [13,18]. The Chinese version of CHBMS showed good reliability with Cronbach’s α coefficients of 0.881 for the total scale and 0.801 to 0.944 for all the dimensions [21].

### Data collection

The study was conducted at an outpatient clinic were recruited from a first-class tertiary hospital in Guangzhou. The Department of Colorectal Surgery is one of the largest CRC medical research centers in China, with the largest known cohort of hereditary CRC patients, including nearly 500 hereditary CRC patients and more than 12,000 blood relatives. This hospital represents the highest level in the field of hereditary cancer in China. After obtaining the consent of the Nursing Department and the chief nurse, one researcher was responsible for the data collection process. The investigator adopted a face-to-face survey with the participants. Finally, 530 of 561 participants conformed to the criteria finished the survey (94.47%).

## Data analysis

We executed all analysis in SPSS (Version 26.0, USA). Continuous variables were described using mean and standard deviations, while frequencies and percentages were used to describe the categorical variables. The χ2 test was used to evaluate the correlation between demographic and disease variables and malignancies early detection. Differences between subgroups in malignancies early detection associated with CRC knowledge, screening attitude and health beliefs were determined using t-test and analysis of variance test. Binary logistic regression model was performed with all variables that were significant in the univariate analysis associated with malignancies early detection. Odds ratios and 95% confidence intervals were calculated for the predictors in the models. Discrimination was evaluated using the area under the receiver operating characteristic (ROC) curve (AUC) (C-index of the model), which indicated the model’s ability to discriminate between patients with a high versus low probability of returning to work. AUC values of 0.70–0.79 were considered to indicate acceptable discrimination, 0.80–0.89 as excellent and 0.90 or more as outstanding discrimination. Finally, the explained variance was calculated in terms of Nagelkerke’s R2 value. All p-values are two-sided and a p-value of < 0.05 was considered statistically significant.

## Ethical considerations

The clinical research ethics committee of Sun Yat-sen University Cancer Center (no.B2021-185–01) approved this research. The purpose and content of this study were interpreted to the individuals. Confidentiality and anonymity were assured. Each participant was assured that his treatment would not be influenced by rejecting or withdrawal at any time. All the completed forms were placed in sealed envelopes and stored in a secure place accessible only to the researcher. Their materials were just used for the present research and will be destroyed once related results are published.

## Results

### Sample characteristics

A total of 561 FDRs of 312 CRC patients were invited to participate in the study, and all enrolled participants agreed to complete the questionnaires. However, 31 individuals completed less than 80% of the questionnaire. As a result, the completion rate was 94.47%, and 530 FDRs from 294 hereditary CRC patients were ultimately included in the study. No significant differences were found in the demographic characteristics between the respondents and non-respondents.

The age of the participants in this study ranged from 18 to 70 years, with a mean (SD) age of 36.22 (12.45) years. Participants (n = 530) were partly female (50.6%) and married (63.4%), most were middle school education or above (96.2%). Nearly half of the FDRs (49.5%) had a family income with 3000–7000 Yuan. Urban residents accounted for 74.4% and 24.9% of participants who smoked. The majority of participants (54.0%) were children of the probands, 39.2% were their siblings, and 6.8% were parents. In all the hereditary CRC families, 2–8 relatives had been diagnosed with CRC or relative cancer at the earliest age 41.18 ± 11.65. Table 1 shows more information. There were 294 index patients aged 48.86 ± 11.73 (range from 14 to 73), men accounted for 65.3%, they were diagnosed with single primary CRC (75.0%), multiple primary CRCs (9.5%), CRC combined with other extraintestinal tumors (11.5%) or clinically diagnosed FAP (4.0%). The vast majority of the patients were diagnosed after the onset of symptoms, with only 47 (8.9%) ones were diagnosed through early detection and surveillance for malignancies. 98 (48.0%) of the patients were delayed for CRC diagnosis. Additionally, we separately categorized the age groups, using 25 years as the cutoff. There were 73 individuals aged 24 or younger, of whom 4 (5.5%) had undergone colonoscopy screening. For those aged 25 or older, there were 457 individuals, with 118 (25.8%) having undergone colonoscopy screening. The chi-square test revealed a significant difference between the two groups (P < 0.001). More details were showed in Table 1.

**Table 1:** Univariate analysis of demographic characteristics and cancer screening (N = 530).

Variable	Number of subjects (n,%)	Ever underwent cancer screening (n = 122)	Never cancer screened (n = 408)	Statistical value	*p*
**Age**		42.38 ± 13.89	34.38 ± 11.37	6.462^a^	<0.001
＜40	366(69.1)	64	302	20.431^b^	<0.001
≥40	164(30.9)	58	106		
**Gender**				7.894^b^	0.005
Male	262(49.4)	74	188		
Female	268(50.6)	48	220		
**Education level**				24.981^b^	<0.001
Primary school	20(3.8)	0	20		
Middle school/technical secondary school	232(43.8)	60	172		
College/undergraduate	254(47.9)	52	202		
Postgraduate	24(4.5)	10	14		
**Marital status**				32.604^b^	<0.001
Single	194(36.6)	18	176		
Married	336(63.4)	104	232		
**Family monthly income per capita (CNY)** ^c^				2.752^b^	0.738
< 3000	109 (20.6)	24	85		
3000 ~ 4999	154 (29.1)	39	115		
5000 ~ 6999	108(20.4)	19	89		
7000 ~ 10000	80(15.1)	20	60		
>10000	79(14.9)	20	59		
**Medical insurance**				1.443^b^	0.230
Urban health insurance/ free medical service	314(59.2)	78	236		
New Rural Cooperative Medical System/No health insurance	216(40.8)	44	172		
**Place of residence**				7.135^b^	0.008
City	394(74.3)	102	292		
Rural area	136(25.7)	20	116		
**Employment status**				0.694^b^	0.405
Employed	384(72.5)	92	292		
Unemployed/retired	146(27.5)	30	116		
**Chronic disease history**				29.686^b^	<0.001
Yes	76(14.3)	36	40		
No	454(85.7)	86	368		
**Index patient’s diagnosis**				17.665^b^	<0.001
Single primary intestinal cancer	398(75.1)	74	324		
Multiple primary cancers	132(24.9)	48	84		
**Number of relatives with colorectal cancer**				0.723^b^	0.395
≤ 4 people	424(80.0)	94	330		
> 4 people	106(20.0)	28	78		

^a^t-value,

^b^Chi-square value,

^c^10000 CNY is approximately US$1568.

### Knowledge, attitudes and health belief among FDRs of patients with hereditary CRC

The characteristics of total knowledge score, knowledge score on symptoms of disease, factors for disease and detection and diagnostic methods were showed in Table 2.

**Table 2:** Univariate analysis of knowledge, health beliefs and cancer screening (N = 530).

Variable	Mean (SD)	cancer screening (Mean (SD))		T *(P)*
		Ever (n = 122)	Never(n = 408)	
**Knowledge**	13.58(7.40)	16.08(6.31)	12.83(6.23)	5.015(<0.001)
disease symptoms	5.30(2.41)	6.21(2.52)	5.02(2.31)	4.868(<0.001)
disease-related factors	5.77(3.02)	6.84(3.49)	5.45(2.79)	4.555(<0.001)
screening and diagnostic methods	2.51(1.97)	3.02(2.30)	2.35(1.83)	3.329(<0.001)
**Health beliefs**	3.37(0.36)	3.42(0.29)	3.24(0.36)	5.100(<0.001)
Perceived benefits	3.73(0.63)	3.99(0.59)	3.65(0.63)	5.302(<0.001)
Health motivation	3.69(0.57)	3.84(0.51)	3.62(0.59)	3.217(<0.001)
Perceived self-efficacy	3.54(0.59)	3.81(0.57)	3.46(0.87)	5.863(<0.001)
Perceived severity	3.14(0.63)	3.27(0.52)	3.12(0.66)	2.400(0.017)
Perceived barriers	3.02(0.68)	3.04(0.73)	3.02(0.67)	0.338(0.736)
Perceived susceptibility	2.80(0.70)	3.11(0.64)	2.84(0.71)	3.817(<0.001)

The mean (SD) health belief score of FDRs of patients with hereditary CRC was 121.36 (13.00), with a mean (SD) score of 3.37 (0.36) for each item. Among the various dimensions, the perception of the benefits of early malignancy detection had the highest scores. The scores for the remaining dimensions, from highest to lowest, were as follows: health motivation, perceived self-efficacy, severity, barriers, and susceptibility (Table 2).

Table 3 shows the scores of attitudes towards early detection and surveillance for malignancies. Most of the FDRs had a positive attitude towards malignancies early detection, the proportion of positive answer of the issues range from 64.2% ～ 81.1% respectively.

**Table 3:** Attitudes of cancer screening (N = 530).

Categories	N (%)	Ever underwent cancer screening (n = 122)	Never cancer screening (n = 408)	Statistical value	*P*
cancer is preventable	400(75.5)	112(91.8)	288(70.6)	22.834	<0.001
early cancer is curable	422(79.6)	115(94.3)	307(75.2)	20.934	<0.001
cancer screening can help to find early cancer	430(81.1)	117(95.9)	313(76.7)	22.584	<0.001
cancer screening can help to prevent cancer	388(73.2)	85(69.7)	303(78.1)	1.010	0.315
cancer screening is beneficial	400(75.6)	112(91.8)	288(70.6)	24.761	<0.001
cancer screening is important	394(74.3)	117(95.9)	277(67.9)	38.625	<0.001
One needs cancer screening even if feel healthy	340(64.2)	115(94.3)	225(55.1)	62.482	<0.001
willingness to self-finance cancer screening	361(68.1)	109(89.3)	252(61.8)	32.891	<0.001

N/n: the individuals agreed with the opinion with positive attitudes of cancer screening.

### Early detection for malignancies and barriers among FDRs of patients with hereditary CRC

**Fig 1:**
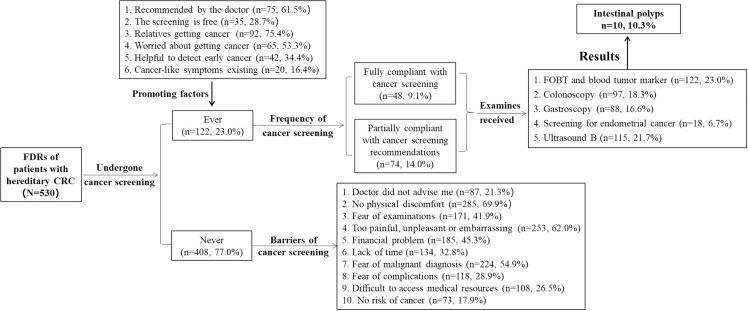
Cancer screening of the participants.

Of all the participants, 122/530(23.0%) completed malignancies early detection. Only 48(9.1%) participants reported they had accepted malignancies early detection annually according to the doctors’ instructions. 122(23.0%) participants reported they had accepted FOBT and blood tumor marker. The section who had undergone colonoscopy, gastroscopy, screening for endometrial cancer and Ultrasound B is 97(18.3%), 88(16.6%), 18/268(6.7%) and 115(21.7%), respectively. The reasons that urged them to undergo malignancies early detection contained relatives getting cancer, recommended by the doctor and so on(Fig 1). All FDRs who had never accepted malignancies early detection reported the probable barriers of no malignancies early detection. More details were showed in Fig 1.

### Predictive factors of early detection for malignancies among FDRs of patients with hereditary CRC

According to the univariate analysis, age, gender, education level, marital status, place of residence, chronic disease history, index patient’s diagnosis, the attitudes of cancer and malignancies early detection, knowledge and health beliefs were associated with the malignancies early detection among FDRs of patients with hereditary CRC (Tables 2 and 3).

**Fig 2:**
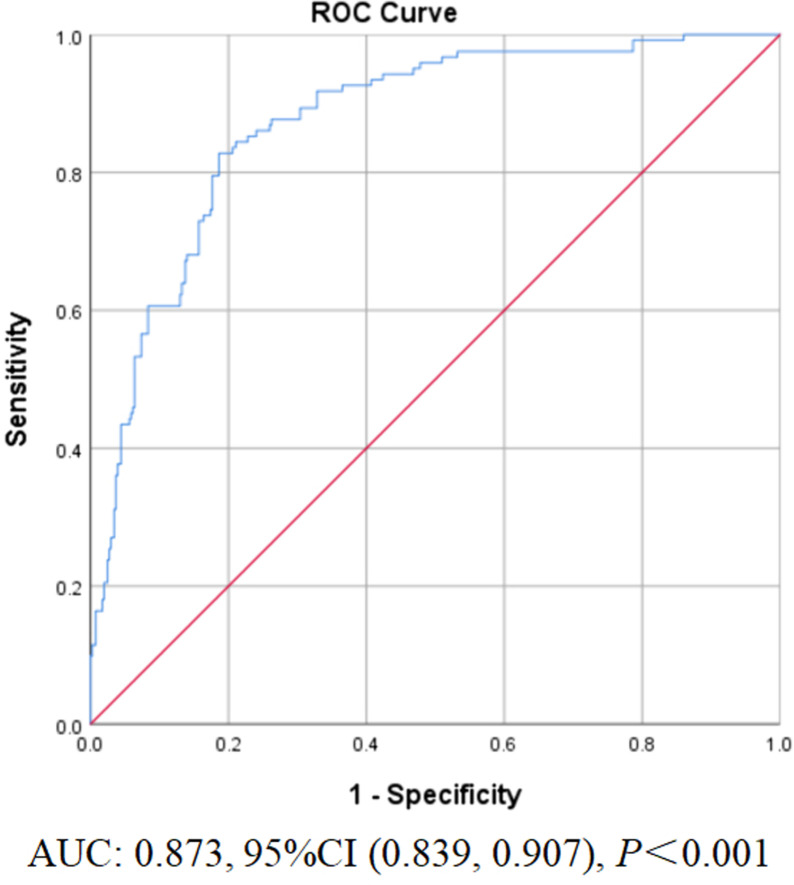
Bootstrapped receiver-operating characteristic (ROC) curves for the model prediction of cancer screening.

Binary logistic regression analysis was conducted to identify predictive factors of malignancies early detection among FDRs of patients with hereditary CRC, as presented in Table 4. The predictive factors included knowledge score, sex, age, marital status, chronic disease history, diagnosis of index patient, attitude about “cancer is preventable” and “one needs malignancies early detection even if feel healthy”, perceived susceptibility, perceived self-efficacy (p < 0.05). Participants who were male, over 40 years old, married, with history of chronic disease, the corresponding index patient with multiple primary cancers, with the positive attitude of cancer is preventable and one needs malignancies early detection even if feel healthy, were more likely to have attended malignancies early detection compared with those in the reference group, the OR is showed in Table 4. Additionally, with each single-unit increase in knowledge score, the probability of FDRs undergoing malignancies early detection increased 1.117 times. In contrast, for each 1-unit increase in perceived susceptibility and perceived self-efficacy, the probability of FDRs undergoing malignancies early detection increased 1.106 times and 1.244 times respectively on the Champion’s health belief model scale. The Cox & Snell coefficient of determination (R^2^) was 36.9%, whereas the Nagelkerke coefficient of determination (R^2^) was 55.9%. The AUC of the model was 0.873 (95% CI 0.839 to 0.907). The bootstrapped ROC curve is shown in Fig 2.

**Table 4:** Predictive factors of cancer screening in FDRs of hereditary CRC patients (N = 530).

Variable	β	SE	Wald	*P*	OR	95% CI
**Knowledge score**	.110	.023	22.488	<0.001	1.117	1.067 ~ 1.169
**Sex**						
Male (reference)						
Female	-1.410	.316	19.979	<0.001	.244	.131 ~ .453
**Age**						
<40 years (reference)						
≥40 years	1.532	.321	22.762	<0.001	4.627	2.466 ~ 8.683
**Marital status**						
Single(reference)						
Married	1.339	.358	13.966	<0.001	3.815	1.890 ~ 7.698
**Chronic disease history**						
No (reference)						
Yes	1.080	.365	8.752	.003	2.945	1.440 ~ 6.024
**Diagnosis of index patient**						
Single primary colorectal cancer (reference)						
Multiple primary cancers	1.056	.324	10.608	.001	2.876	1.523 ~ 5.432
**Cancer is preventable**						
No (reference)						
Yes	1.225	.482	6.459	.011	3.405	1.324 ~ 8.760
**One needs cancer screening even if feel healthy**						
No (reference)						
Yes	2.802	.504	30.862	<0.001	16.477	6.131 ~ 44.279
**Perceived susceptibility**	.101	.044	5.263	.022	1.106	1.015 ~ 1.206
**Perceived self-efficacy**	.218	.057	14.723	<0.001	1.244	1.113 ~ 1.390

Model *χ*^2^** = **327.906, df** = **7, P<0.01, Cox & Snell R^2^** = **36.9%, Nagelkerke R^2^** = **55.9%.

Abbreviations: β, regression coefficient; CI, confidence interval; CRC, colorectal cancer; FDRs, first-degree relatives; OR, odds ratio.

## Discussion

To our knowledge, there is a lack of data evaluating the effectiveness of such initiatives on early detection of malignancies, and this is the first study to describe malignancies early detection and its predictors among FDRs of patients with hereditary CRC in China. FDRs of patients with hereditary CRC, as a high-risk group for carcinomas, should begin early detection and surveillance at an early age to detect and treat precancerous lesions in a timely manner, thereby preventing progression to cancer. Our results indicated that, despite being recommended for early detection and surveillance, the detection rate among FDRs was only 23.0%, which is significantly lower compared to the effective screening rate of 85% [15]. We found that some participants underwent only part of the recommended examinations. The highest participation rates were observed for the fecal occult blood test (FOBT) and blood tumor marker testing, while the detection rate for endometrial cancer was very low. This distribution is similar to findings from investigations conducted in the United States [22]. Perhaps due to examine of FOBT and blood tumor marker are easy-to-apply, low-cost and reliable. The completing rate of colonoscopy is 18.3%, Which is similar to the screening rate among other high-risk populations and general in China with 18.6% and 18.9%, respectively [23,24]. But we should stress that colonoscopy is the best strategy for early diagnosis of CRC. There have been surveys of the early detection and surveillance for malignancies rate of FDRs in various countries to date. An Australian study among FDRs reported a screening rate on cancer of 25% to 49% [25]. In the United States, where cancer screening is relatively well established, 45% of 2470 FDRs had participated in colonoscopy [26], which indicate that the screening rate in this population from China is very low and should be improved. In addition, almost half of new cases and deaths from CRC are in Asia, according to regional estimates; few studies in China and Korea found that 18.9% ～ 31.9% adults intended to adhere to screening of CRC [11,24]. However, to our knowledge, rare study has focused on early detection and surveillance behavior among FDRs of patients with hereditary CRC in Asia.

In addition, this study explored the motivations and barriers to early detection of malignancies in FDRs of patients with hereditary CRC. As shown in the results, the motivations for early detection and surveillance included recommendations from physicians, free screening, having relatives with cancer, concerns about developing cancer, the potential to detect cancer early, and the presence of cancer-like symptoms. Similar findings have been reported by scholars in other countries. Stoffel [22] reported that it is not a surprise that individuals who had some close relatives with relative young-onset cancer were more likely to accept appropriate malignancies early detection. Previous study from America also indicated that doctors’ recommendations may be one important target for intervention to improve the rate of malignancies early detection [22]. Otherwise, in regard to the barriers of malignancies early detection, we found ‘No physical discomfort’ was the most common barrier for uptake screening, which was consistent with previous studies [27,28]. This implies that participants mistakenly are convinced that malignancies early detection is only required on occasion of symptoms or feeling ill. We should let the objects understand the fact that disease only becomes symptomatic when it is at an very advanced stage [15]. Moreover, ‘Too painful, unpleasant or embarrassing’ was another major reason for not participation in malignancies early detection, which implies that better communication with participants on the relative detecting would be conducted to remove the perception of fear and embarrassment. In addition, “Fear of malignant diagnosis” is the third reason of no cancer detecting, it may be that cancer is a taboo topic in Chinese culture. The notion of detecting an asymptomatic disease by screening does not exist in Chinese traditional beliefs [29]. It is essential to design culturally tailored education and interventions to reduce negative attitudes toward cancer and increase awareness of the importance of screening for this preventable disease. Other barriers identified suggest that physicians should play a more active role in providing information on early detection of malignancies to FDRs of patients with hereditary CRC, enhancing their understanding of cancer screening. Furthermore, a “one-size-fits-all” approach is not suitable for early detection in hereditary CRC. A detailed review of the patient’s family history should be incorporated into discussions about early detection and surveillance. Additionally, it is crucial to conduct awareness campaigns to capture public attention, correct misconceptions, and address psychological barriers.

We also explored the predictive factors for early detection of malignancies among FDRs of patients with hereditary CRC. The results of the binary logistic regression analysis identified several demographic and health-related characteristics, knowledge score, perceived susceptibility, and perceived self-efficacy as significant predictors. The findings showed that males, individuals over 40 years old, and married individuals were more likely to participate in early detection. It may be because of the guidelines recommendation that people older than 40 years and mail should be a priority group for routine malignancies early detection [2,16]. And individuals married may be urged by the family members to undergo screening, as some evidence suggests that socially disadvantaged groups were less likely to complete malignancies early detection [30]. But as the age of onset of hereditary cancer usually before 40 years old, so we should pay attention to the young group of their FDRs [31]. Previous studies indicated that individuals who had never seen a doctor within the past several years may have insufficient access to medical resources and were less aware of information on malignancies early detection [22], similar to the result in this study about ones with history of chronic disease and the corresponding index patient with multiple primary cancers. This study discovered that those with better knowledge and attitude about “cancer is preventable” and “one needs malignancies early detection even if feel healthy” more preferred to undergo malignancies early detection. This finding indicated that the participants correctly estimating their risk of cancer and aware of the importance of malignancies early detection may more likely complete detecting. Wong *et al* [32] also found that participants with better knowledge on screening were more inclined to perform malignancies early detection. It is, therefore, urgent to plan health education interventions to correct public misperceptions of self-risk of developing cancer and to emphasise the significance of malignancies early detection. The last two predictors were from the HBM, perceived susceptibility and perceived self-efficacy. Individuals with higher level of susceptibility were more likely to have performed malignancies early detection, which was consistent with the HBM [18,25]. And as Choi *et al* [33] noted that ones with a greater perceived risk of cancer were also significantly more likely to complete screening than those with lower perceived risk. Perceived susceptibility and self-efficacy means the one’s assessment is positive regarding the possibility of developing a cancer and his ability to perform malignancies early detection. A systematic review also indicated that perception of cancer risk and self-efficacy was a predictor of screening behavior [18]. These information suggest that providing intervention to FDRs to improve their understanding and awareness that they are at a high risk for relative cancer will be a key way to improve screening rates, and health professionals will play important roles in this process [34]. In one study, although respondents acknowledged the threat of cancer and the importance of screening, they believed they were inevitably going to develop cancer, rather than thinking it could be controlled. As a result, they viewed early cancer screening as unnecessary and ineffective, which led to their refusal to participate in early detection [35]. Indeed, a risk counseling intervention focused on family history found that family-oriented interventions could significantly increase malignancies early detection rate [36]. Therefore, healthcare professionals should provide tailored health education to emphasize the importance of screening and individualized risk assessments for target FDRs who accompany hereditary CRC patients during medical treatment. This approach can enhance their perceived susceptibility to cancer and improve their self-efficacy regarding early detection and surveillance, ultimately increasing the likelihood of malignancies being detected early.

## Strengths and limitations

Our findings indicate that FDRs of patients with hereditary CRC have limited knowledge, some misconceptions, and face barriers related to early detection, with many being unaware of the need for such screening. Consequently, the early detection rate among this group should be improved. Additionally, the study has identified key predictors of early detection, which can inform future research and clinical practice in this area.

This study has several limitations. First, this study was completed in a selected population who had direct or indirect contact with a hereditary clinic and were willing to spend 30-minutes finished a self-reported measurement. Consequently, these were highly motivated ones who were aware of their family history of cancer. Second, the outcome of malignancies early detection was assessed based on individuals’ self-reports of detections and we could not obtain medical records confirmation for their reports. However, previous scholars have verified the reliability of ones’ self-reports of malignancies early detection [37,38] and abundant studies, including the U.S. National Health Interview Surveys (NHIS), collect information on practices of malignancies early detection by subject interviews.

Third, we did not document the specific type of hereditary cancer predisposition syndrome (e.g., Lynch syndrome or FAP) to which each index patient and their FDRs belonged. However, different syndromes are associated with markedly different cancer risks, surveillance recommendations, and clinical outcomes. For instance, endometrial cancer risk is significantly increased in Lynch syndrome but not in FAP, and the recommended age to initiate colonoscopy varies accordingly. Future studies should incorporate genetic or clinical confirmation of hereditary cancer syndromes to allow for more specific risk stratification and tailored recommendations.

Fourth, the respondents were all recruited from one hospital in Guangzhou, so it might jeopardize the generalizability of the results. But this hospital has the largest known cohort of hereditary colorectal cancer patients and represents the highest level in the field of hereditary cancer in China, so the result is deserve to be attention. Fifth, we conducted a cross-sectional exploratory design in present study. Prospective dynamic, multi-center follow-up studies could be carried out in the future. Last, the influence of clustering from the same family wasn’t considered. But most of the respondents in our study come from different families eventually. The predicting factors didn’t contain the support system of malignancies early detection, which may have some specific effect on malignancies early detection. Thus, future study must consider the role of family and environmental factors on malignancies early detection. A machine learning-based prediction method may be optimized way to explore more comprehensive, deeper predictors of malignancies early detection.

Additionally, our study raises questions concerning the management of FDRs from a hereditary cancer family in oncology practice:(1) What are the potential drawbacks among relatives of patients with hereditary cancer when seeking screening services in terms of individual, environment and context, and how can we handle such challenges? (2) How can cancer-screening services be improved further to extend to relatives of patients with hereditary cancer?

## Conclusions

Despite it is known that early detection of malignancies is crucial for FDRs of patients with CRC, this study found that the screening rate among this population remains low in China. Our research identified key predictors of early malignancy detection among FDRs of hereditary CRC patients, emphasizing the need for health professionals to enhance awareness, perceptions of susceptibility, and address barriers to early detection at the individual, family, and societal levels.

Additionally, our findings suggest that several strategies could improve early detection rates. First, the government should implement targeted educational campaigns, leveraging web-based tools to disseminate scientific information on cancer prevention. Second, physicians play a critical role in promoting early detection by offering professional recommendations. Nurses, as a key component of the healthcare workforce, should also be trained to actively contribute to improving knowledge, perceptions, and early detection practices among high-risk populations.

These insights pave the way for developing interventions aimed at improving early malignancy detection and offer a pathway for expanding the roles of specialist nurses in genetic clinical practice.
